# Case report: positive pitfalls of PSMA PET/CT: diagnostic challenges in degenerative bone lesions including MODIC type 1

**DOI:** 10.3389/fnume.2024.1451848

**Published:** 2024-08-02

**Authors:** Hicham Moukaddam, Ghida El Saheb, Nadine Omran, Nour El Ghawi, Alain Abi Ghanem, Mohamad Haidar

**Affiliations:** Department of Diagnostic Radiology, American University of Beirut, Beirut, Lebanon

**Keywords:** PSMA, PET/CT, prostate cancer, bone lesion, MODIC type 1, case report

## Abstract

Prostate-specific membrane antigen (PSMA) positron emission tomography/computed tomography (PET/CT) is an imaging technique that has demonstrated high sensitivity and specificity in detecting prostate cancer and its metastasis, especially in the bones. This case describes a 60-year-old man who presented for increased prostate-specific antigen (PSA) level and underwent [^68^Ga]gallium-PSMA-11 PET/CT imaging for better disease assessment. ^68^Ga-PSMA-11 PET/CT revealed numerous radiotracer-positive lesions in both prostate lobes with associated sclerotic lesions on L4 and L5, but only L5 showed increased radiotracer avidity raising the possibility of metastasis. Magnetic Resonance Imaging (MRI) raises the possibility of aggressive MODIC type 1 lesion vs. infectious/inflammatory process. A biopsy of the radiotracer avid area was performed and showed no evidence of metastasis. The final diagnosis was aggressive MODIC type 1, in keeping with the false positive result of ^68^Ga-PSMA-11 PET/CT. This example demonstrates the possible limitations of ^68^Ga-PSMA-11 PET/CT, particularly in detecting bone metastases, and emphasizes the need for cautious interpretation and additional study to improve its diagnostic accuracy. Understanding and resolving these limitations is critical for increasing the accuracy of PSMA PET/CT in prostate cancer management.

## 1 Introduction

Prostate-specific membrane antigen (PSMA) is a non-secreted transmembrane protein expressed in prostate tissue and tumor-related blood vessels. PSMA uptake has been observed in a variety of tumors and benign diseases (1, 2). Nevertheless, PSMA has shown the most promise in the detection and staging of prostate malignancy and is now considered the optimal imaging technique in the diagnosis of prostate cancer (3, 4). In a meta-analysis of seven studies compromising 389 patients undergoing initial diagnosis of prostate cancer, PSMA positron emission tomography/computed tomography (PET/CT) demonstrated a pooled sensitivity and specificity of 0.97 and 0.66, respectively, with a positive likelihood ratio of 2.86 and negative likelihood ratio of 0.05 demonstrating that PSMA PET/CT is a useful technique for excluding malignancy in patients with clinical suspicion of prostate cancer, thus avoiding unnecessary biopsies (5).

Prostate cancer is known to spread to cortical bone and bone marrow, contributing to the majority of prostate cancer-related fatalities. While bone scans are used to detect osteoblastic skeletal metastases, they lack specificity to distinguish between benign and malignant lesions (6). Combining these bone scans with other biomarkers, notably prostate-specific antigen (PSA) levels can aid in detecting metastasis (BM), but remains insufficient to predict the development of bone metastasis accurately (7). Nevertheless, PSMA PET/CT has demonstrated excellent abilities to detect bone metastasis with a sensitivity ranging from 80% to 100% and a specificity ranging from 95.6% to 100%, outperforming bone scans and Magnetic Resonance Imaging (MRI) (8). PSMA PET relies on the PSMA Reporting and Data System (RADS) rating, maximum standardized uptake value (SUVmax), and SUVmax ratio for the lesion to the blood pool to improve diagnostic accuracy in diagnosing metastasis (9).

Although many studies have demonstrated PSMA PET/CT accuracy in detecting metastatic bone lesions, this case demonstrates an instance in which ^68^Ga-PSMA-11 PET/CT incorrectly diagnosed a bone lesion as metastatic raising concerns about PSMA PET/CT’s specificity in detecting bone metastases.

This is the case of a 60-year-old male, previously healthy, who presented following a high PSA level of 6.7 ng/ml on his annual check-up tests.

## 2 Case

**Figure 1:**
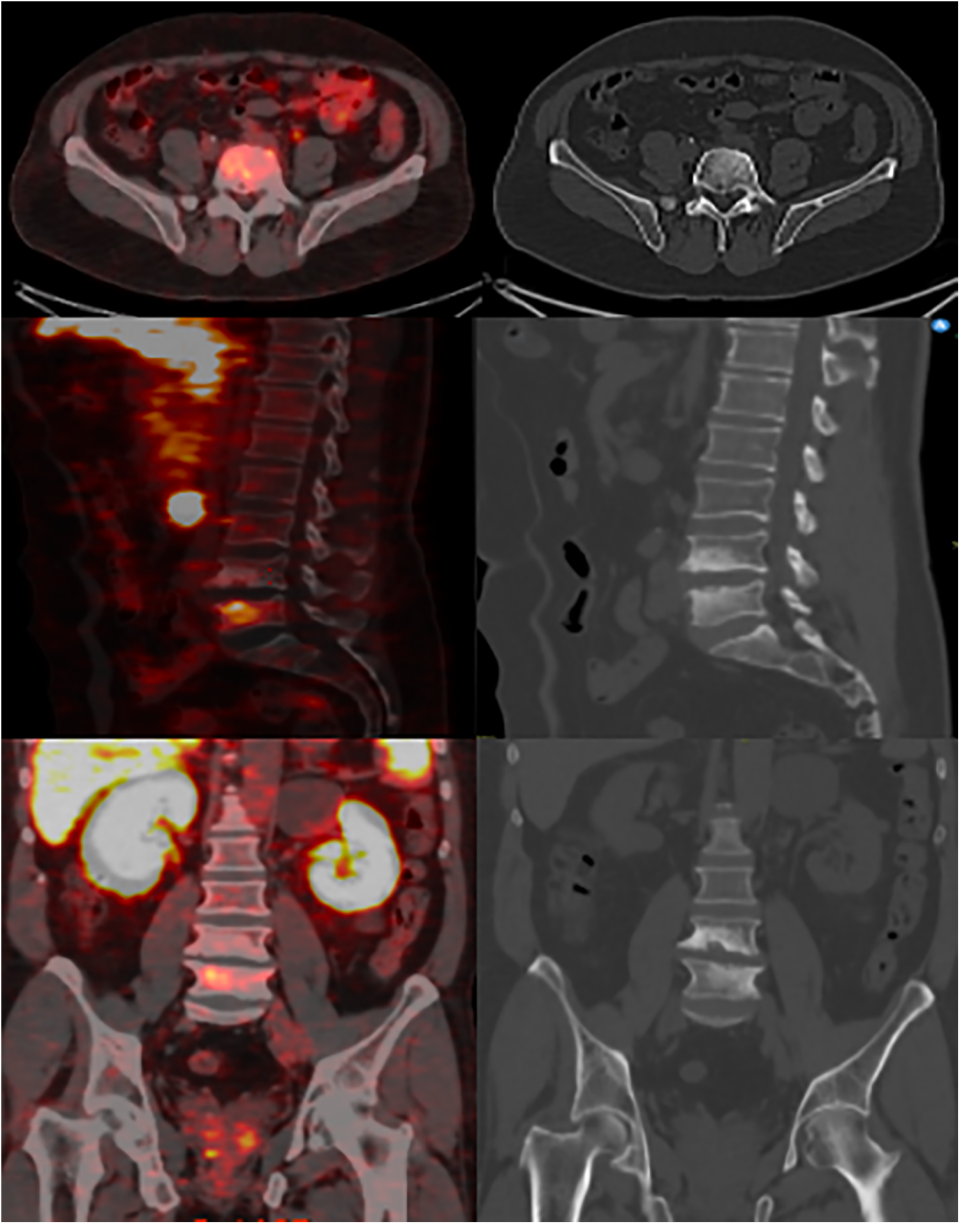
^68^Ga-PSMA-11 PET/CT images with multiplanar reformats show ill-defined endplate sclerosis, irregularities, and small erosions around the L4-L5 intervertebral disc with increased focal radiotracer uptake at the L5 vertebral body.

**Figure 2:**
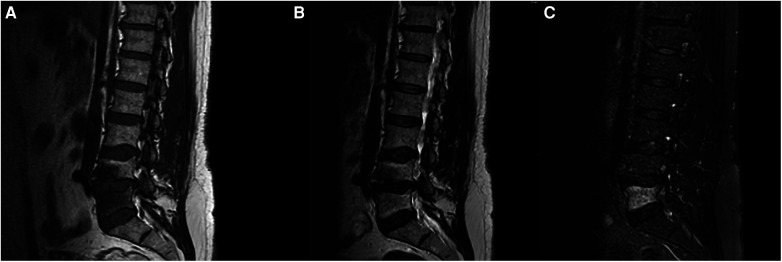
MRI of the lumbar spine shows advanced degenerative disc changes at L4-L5 level with endplate irregularities and small schmorl's nodes seen on (**A**) T1 and (**B**) T2 weighted-images as well as bone marrow signal abnormality at L5 vertebral body best appreciated on (**C**) STIR images.

**Figure 3:**
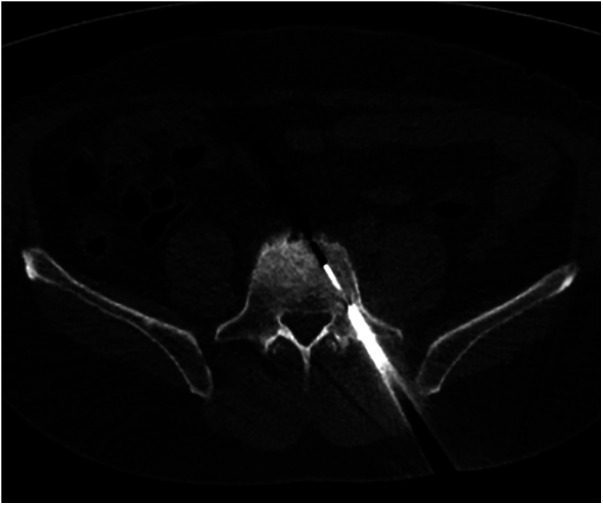
CT-guided percutaneous core biopsy of the sclerotic and radiotracer-avid area at the L5 vertebral body through a transpedicular approach.

**Figure 4:**
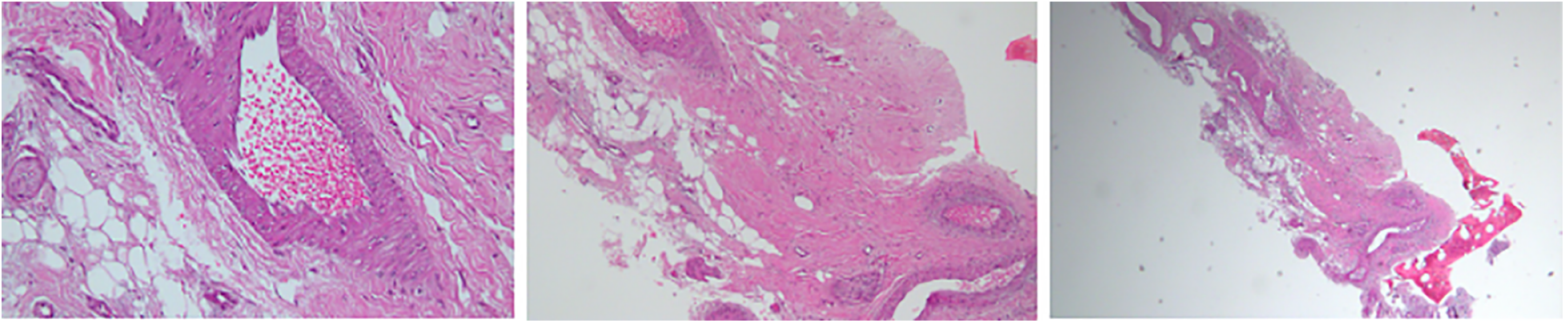
Fragments of bone and bone marrow. No inflammatory cells. Negative for malignancy.

A 60-year-old male, previously healthy, was found to have a high PSA level of 6.7 ng/ml on his annual check-up tests. The patient reported no back pain, urinary symptoms, hematuria, weight loss, fatigue, or other systemic symptoms. The patient underwent a prostate Magnetic Resonance Imaging (MRI) that revealed a 1 cm prostatic nodule in the left peripheral zone characterized as Prostate Imaging Reporting and Data System (PIRADS) IV with no suspicious pelvic lymphadenopathy. To further evaluate the prostatic lesion, a biopsy was performed revealing adenocarcinoma in both the left midgland (Gleason 6, grade I) and the left base (Gleason 7, grade II). ^68^Ga-PSMA-11 PET/CT revealed numerous radiotracer-positive lesions in both prostate lobes- the left prostate SUVmax was 10.7 and the right prostate SUVmax was 4.3- with associated sclerotic lesions on L4 and L5, but only L5 showed increased radiotracer avid with an SUVmax of 6.5 raising the possibility of metastasis (Figure 1). In light of these findings, an MRI was recommended to determine the nature and extent of the lesion in the vertebral body. The MRI of the lumbar spine did not show any signs of bony metastasis but raised the possibility of an aggressive MODIC type 1 lesion vs. an infectious/inflammatory process (Figure 2). An infectious work-up was done to exclude an underlying infectious process similar to imaging appearances. The patient had no fever and did not report back pain. Moreover, inflammatory markers were normal, and both blood and lumbar spine cultures showed no abnormalities. A CT-guided biopsy was performed on the bone lesion at L5 of the radiotracer avid area, and no evidence of metastasis, infection, or inflammation was found on histology (Figures 3, 4). The final diagnosis indicated an aggressive MODIC type 1, raising concerns about false positive results from the ^68^Ga-PSMA-11 PET/CT. MODIC type 1 lesions are considered aggressive when findings such as endplate erosion and extraspinal inflammation are present (10). In our case, we observed endplate erosions on CT and inflammation/bone marrow edema on MRI, indicative of aggressive MODIC type 1.

## 3 Discussion

This case describes an unusual incidence of false positive findings in detecting bone metastasis with ^68^Ga-PSMA-11 PET/CT, a modality known for its high specificity and positive predictive value in prostate cancer detection and staging (6–8). Even in patients with low serum PSA, bone metastases are common; thus, PSMA PET/CT is a promising tool to assess bone metastases and provide accurate bone staging (11).

While ^68^Ga-PSMA-11 PET/CT hinted at probable bone metastases in the L5 vertebral body, MRI results ruled out the presence of metastatic lesions. However, it raised concerns about the presence of either an aggressive MODIC type 1 lesion or an infectious/inflammatory process. MODIC type 1 vertebral endplate changes is a classification for vertebral body endplate MRI signal changes. It appears as a hypointense signal on T1 and a hyperintense signal on T2 in the marrow near the vertebral body endplates. These modifications are dynamic and can progress to Modic type 2 alterations over time. The causes of MODIC type 1 are unclear. Proposed theories include severe injury, localized inflammation caused by proinflammatory mediators, and low-grade bacterial infection (10). Although the relationship between MODIC changes and radiotracer uptake in bone scintigraphy has not been examined thoroughly, single photon emission computed tomography (SPECT) findings suggest that MODIC type 1 changes are linked to higher tracer uptake, which may reflect higher bone turnover in the affected region (12). The increased uptake can also be related to the increased lesion's vascularization (13). It is important to note that MODIC type 1 lesions may not exhibit radiotracer uptake on Ga-PSMA PET scans (14). To our knowledge, this is the first reported case where a MODIC type 1 lesion shows radiotracer uptake on ^68^Ga-PSMA.

Previous studies have shown that PSMA PET/CT scans may produce false positive results, particularly in solid organs like the liver and the spleen, and in bones like the ribs. False positive diagnosis of liver metastases, before or after therapy, has been highlighted in many cases (15). Moreover, false-positive PSMA uptake in benign hemangiomas, including splenic hemangiomas, has also been reported (16). Various factors can be considered when distinguishing between benign and malignant lesions on PSMA PET. These factors include PSMA RADS, SUVmax, and the SUVmax ratio for the lesion to the blood pool. A lesion-to-blood pool SUVmax ratio higher than 2.2 is a reliable parameter for supporting image interpretation. It has been shown to offer improved lesion detection and specificity compared to visual interpretation using PSMA RADS (9).

Fractures, degenerative changes, geodes, Schmorl's nodes, fibrous cortical defects, fibrous dysplasia, Paget's disease, vertebral hemangiomas, and bursitis are examples of musculoskeletal disorders that might result in false positive PSMA PET/CT imaging results (17). Moreover, many studies focused on rib and osteophyte metastatic lesions and investigated the effectiveness of PSMA PET/CT in accurately determining whether a lesion is benign or malignant. One of the challenges doctors face is that single rib lesions seen on staging PSMA PET/CT scans for prostate cancer often produce false positive results (18, 19). The study showed that most rib lesions with low-intensity uptake are usually benign. While combining CT scan results with a history of trauma can be helpful, it is not always sufficient evidence. It is important to note that relying solely on SUVmax levels is not a reliable way to detect malignancy, as it can lead to false positives (19). For instance, in patients who underwent radical prostatectomy, more than 80% of males with undetectable PSA levels had false positive PSMA avid foci, particularly in skeletal regions including the ribs and pelvis, with nearly all having SUVmax values less than 7. It is critical to recognize this potential issue while analyzing PSMA PET/CT scans to avoid misinterpretation and unnecessary diagnostic procedures (20). Another pitfall of PSMA PET/CT is encountered in Paget disease of the bones. In some cases, PSMA PET/CT imaging has shown moderate-to-intense radiotracer uptake in the shoulder, ribs, iliac bones, and vertebral bodies, indicating possible metastatic involvement. However, histopathology revealed that it was Paget disease. The high radiotracer uptake in Paget disease is most likely due to PSMA overexpression in the highly vascularized and remodeling pagetoid bones (21, 22).

Moreover, the use of hybrid PSMA PET/MRI has been studied, particularly in men who are experiencing recurrence of prostate cancer. This method combines both PET and MRI imaging techniques in a single setting. The MRI component is useful in identifying local recurrence of prostate cancer and in characterizing radiotracer acid soft tissues or bone lesions, which helps differentiate between benign and malignant conditions (23–26). For instance, in patients with biochemical recurrence, 68Ga-PSMA-11 PET/MRI detected recurrent prostate cancer with 100% sensitivity and approximately 70% specificity. Thus, combining PET and MRI increased the detection rate of ^68^Ga-PSMA-11 PET from 63.64% to 79.55% (27).

Finally, this case presents an interesting finding of a falsely positive bone lesion identified on ^68^Ga-PSMA-11 PET/CT imaging. This imaging technique is widely recognized for its high accuracy in detecting bone metastasis in prostate cancer. Thus, the significance of this observation lies in the fact that there is limited literature on cases of false positive lesions detected on PSMA PET/CT before any treatment is given, especially in vertebral lesions. Therefore, this case adds to the literature by providing an example of false positive PSMA PET/CT results in the vertebral body, increasing our awareness of the potential diagnostic pitfalls associated with this imaging modality.

## 4 Conclusion

PSMA PET/CT is an efficient imaging technique for diagnosing and staging prostate cancer. It has shown excellent sensitivity and specificity in detecting bone metastasis. However, false positives should be considered, like in the case of vertebral bone sclerosis. Complementary modalities, such as CT scans and MRI, can provide useful information to aid in diagnosis.

## Data Availability

The original contributions presented in the study are included in the article/Supplementary Material, further inquiries can be directed to the corresponding author.
